# Impact of chronic comorbidities on hospitalization, intensive care unit admission and death among adult vaccinated and unvaccinated COVID-19 confirmed cases during the Omicron wave

**DOI:** 10.1177/26335565231169567

**Published:** 2023-04-29

**Authors:** Marc Simard, Véronique Boiteau, Élise Fortin, Sonia Jean, Louis Rochette, Pierre-Luc Trépanier, Rodica Gilca

**Affiliations:** 1 54470 Institut National de Santé Publique du Québec, Québec, QC, Canada; 2 Département de Médecine Sociale et Préventive, 4440 Université Laval, Québec, QC, Canada

**Keywords:** Comorbidity, COVID-19, surveillance data, omicron, severe complication

## Abstract

**Background:**

Comorbidities are important risk factors of severe COVID-19 complications. Their impact during the Omicron wave among vaccinated and unvaccinated COVID-19 cases is not well documented.

**Purpose:**

The objective of this study was to estimate the association between the number of comorbidities and the risk of hospitalization, intensive care unit (ICU) admission, and death among vaccinated and unvaccinated confirmed adult COVID-19 cases during the Omicron wave.

**Research Design and Study sample:**

We performed a cohort study of COVID-19 adult cases of primo-infection occurring during the Omicron wave, from December 5, 2021 to January 9, 2022 using surveillance database of the province of Québec, Canada. The database included all laboratory-confirmed cases in the province and the related information on 21 pre-existing comorbidities, hospitalization, ICU admission, death related to COVID-19 and vaccination status.

**Analysis:**

We performed a robust Poisson regression model to estimate the impact of the number of comorbidities on each complication by vaccination status adjusted for age, sex, socioeconomic status, and living environment.

**Results:**

We observed that the risk of complication increased for each additional comorbidity in both vaccinated and unvaccinated individuals and that this risk was systematically higher among unvaccinated individuals. Compared with vaccinated individuals without comorbidities (reference group), the risks of hospitalization, ICU admission, and death were respectively: 9X (95% CI [7.77–12.01]), 13X (95% CI [8.74–18.87]), and 12X (95% CI [7.57–18.91]) higher in vaccinated individuals with ≥3 comorbidities; 22X (95% CI [19.07–25.95]), 45X (95% CI [29.06-69.67]) and 38X (95% CI [23.62-61.14]) higher in unvaccinated individuals with ≥3 comorbidities.

**Conclusion:**

Our results support the importance of promoting vaccination in all individuals, and especially those with pre-existing medical conditions, to reduce severe complications, even during the Omicron wave.

## Introduction

Pre-existing comorbidities in COVID-19 cases have been a major risk factor for severe complications, including hospitalization, intensive care unit (ICU) admission, and death, since the beginning of the pandemic.^1^ Numerous studies have found that an increase in the number of medical conditions is associated with an increase in the risk of severe complications.^1–3^ Both vaccination and new variants have had an impact on the severity of the disease among COVID-19 cases.^4,5^ Vaccination has provided protection against severe complications for many severe acute respiratory coronavirus-2 (SARS-CoV-2) variants, including the Omicron variant.^4^

Impact of pre-existing comorbidities during the omicron wave is less well documented than for previous variants. The Omicron variant, first reported in South Africa in November 2021, has spread rapidly worldwide, and cases had been confirmed in more than 57 countries as of December 2, 2021.^6^ In Canada's second largest province (Province of Quebec), the majority of cases were from the Omicron variant during the second week of December (between December 5 and 11, 2021).^7^ The severity of complications among COVID-19 Omicron variant cases was lower, even among unvaccinated cases.^5^ A Danish study using population-based surveillance data reported that an increase in comorbidities remained associated with an increased risk of hospitalization among COVID-19 Omicron variant cases.^8^ However, this study did not assess the potential impact of comorbidities on ICU admission and death.

The aim of this study was to estimate the association between the number of comorbidities and the risks of hospitalization, ICU admission, and death in adults during the Omicron wave, accounting for vaccination status.

## Methods

### Study design and data source

We performed a population-based cohort study using surveillance data including confirmed adult cases of SARS-CoV2 infection during the Omicron wave of the pandemic, from December 5^th^, 2021 to January 9^th^, 2022. We started the study when the proportion of the Omicron variant exceeded 50% among COVID-19 cases and stopped when population-based laboratory testing ceased in the province of Quebec. After January 9, systematic testing was restricted to selected groups such as health care workers and nursing homes residents. The surveillance data file (Trajectoire de santé publique [TSP] registry) includes all cases of infection confirmed by laboratory or epidemiologically in the province of Québec and was linked using a unique identifier to the MED-ECHO preliminary transmission file to obtain information on hospitalization and ICU admission related to COVID-19 and to the Quebec Integrated Chronic Disease Surveillance System (QICDSS) to obtain information on pre-existing medical conditions. The QICDSS is a registry that includes information on all physicians’ claims and diagnostic codes for almost all (>99%) of the Quebec population.^9^ We excluded cases with no information on pre-existing medical conditions, as well as hospital-associated infections, that is an case detected more than one week after hospital admission.^10^ The case date corresponds to the earliest of the following three dates: 1) date of the positive laboratory test, 2) date of symptom onset, 3) date of the beginning of the epidemiological investigation conducted by the public health surveillance team.

### COVID-19 complications

A COVID-19 case was considered hospitalized if at least one acute care hospital stay of at least one day with the COVID-19 diagnostic code (International Statistical Classification of Diseases and Health Related Problems, Tenth Revision [ICD-10-CA] diagnosis code: U07.1) was recorded in the preliminary transmissions of the MED-ECHO file. All COVID-19 related hospitalizations were included, regardless of the reason for admission. Any ICU admission was also recorded in MED-ECHO for these hospitalizations. All death directly or indirectly caused by COVID-19 were collected in the surveillance database during the epidemiological investigations of each of the 18 regional Public Health Departments. The relation of each death with COVID-19 is validated by the regional Public Health Departments on the basis of the forms filled in by the doctors at the time of death^11^ and the information obtained in real time from Quebec registry of deaths^12^ where COVID-19 deaths are entered according to the WHO guidelines.^13^ All complications were followed-up from case date until February 13^th^, 2022 (most up-to-date information at the date of data extraction).

### Comorbidities

Using validated algorithms, the number of comorbidities (0, 1, 2, ≥3) was estimated by the cumulation of 21 pre-existing medical conditions identified from ICD codes entered in the QICDSS for all individuals included in the registry on April 1^st^, 2021^14,15^ (Supplementary files, Table A.1). Those conditions include risk factors (including chronic diseases, their risk factors or symptoms) for complications in individuals with COVID-19^2^ such as cardiovascular diseases, respiratory diseases, mental disorders, obesity and Immune system problem (see Table A1 in Supplementary file). Those conditions do not include some low prevalence diseases associated with COVID-19 complications such as Crohn’s disease or Down syndrome as no algorithm is available to extract such conditions in the QICDSS. Based on the 10 years previous to April 1^st^, 2021, a person was considered to have a medical condition if, at least one diagnostic code was recorded in the hospitalization file or at least two diagnostic codes were recorded in the physicians’ claims database within two years, with at least 30 days between each diagnosis code.^14,15^ For cancer, depression, alcohol and drug abuse conditions, search of diagnostics codes was limited to the previous 5 years.^16^

### Vaccination status

Individuals were considered adequately vaccinated if they had received two vaccine doses (or one Janssen vaccine dose) or a combination of these vaccines with a respected minimal interval between the 2 doses.^17^ In the rest of the text, “vaccinated group” or “vaccinated individuals” wording refer to adequately vaccinated individuals. In contrast, “unvaccinated group” or “unvaccinated individuals” refer to inadequately vaccinated individuals.

### Covariates

Covariates included sex, living environment (private seniors’ residences [RPAs], long-term care facilities [CHSLDs], at home or unknown living environment, others [Including intermediary resources centres]), CDC week (from weeks 2021-49 to 2022-01, January 9^th^, 2022 was included in the 2022-01 week), age and the material and social deprivation indices. Age was calculated on the episode date. The material and social deprivation indices were assigned from the 2016 census data according to the postal code of the place of residence.^18^ Material deprivation included information on income, education and employment status. Social deprivation included information on the proportions of people living alone, single-parent families and people who are separated, divorced or widowed.

### Statistical analysis

We estimated frequency distributions for the number of comorbidities, covariates and complications (hospitalization, ICU admission and death) for all cases included in the cohort and by vaccination status. We then estimated the proportion of cases with complication according to the number of comorbidities, stratified by age and vaccination status. For each of these complications, we finally estimated the relative impact of each additional comorbidity on the risk of each complication controlling for vaccination status with a robust Poisson regression model. Each model (one for each outcome) was adjusted for all covariates, and we added an interaction term between the number of comorbidities and vaccination status to allow comparison of the association between the number of comorbidities and the risk of each complication between vaccination groups. Statistical tests were 2-sided with significance at p < 0.05. SAS 9.4 was used for all analyses.

### Sensitivity and supplementary analysis

We estimated the proportion of cases with each complication within the last two weeks of available data when the proportion of Omicron variant cases was greater than 90%, to ensure a more homogeneous cohort. We estimated the proportion of cases with each complication when including hospital-associated cases. We finally estimated the proportion of cases with each complication stratified by the number of vaccine doses. For each outcome, we estimated adjusted relative risk stratified by age subgroup using robust Poisson regression models including interaction terms with age.

### Ethics

Data linkages and analyses were authorized by the National Director of Public Health (Quebec) under respective provincial public health legislation without requirement for research ethics board review.

## Results

We identified 245,956 confirmed adult COVID-19 cases during the first weeks of the Omicron wave that officially began on December 5^th^, 2021 in the province of Quebec, Canada (Supplementary file, Figure A.1). Respectively 1,7%, 0,3% and 0,5% of cases were hospitalized, admitted to ICU or died due to COVID-19 (Table 1). More than 90% of the cases were adequately vaccinated (79% had received two doses and 12% had received three doses). Cases in the vaccinated group were older (mean age = 43 years; median = 40 years) than the unvaccinated group (mean age = 39 years; median = 36 years) and had fewer complications.

**Table 1.** Description of adults with COVID-19 identified during the Omicron wave (Dec 5^th^ 2021-Jan 9^th^ 2022, Quebec, Canada) stratified by vaccination status (*n* = 245,956).

Variable	Total		Number of comorbidities							
			0		1		2		≥3	
	*n* = 245,956		*n* = 156,016		*n* = 51,367		*n* = 18,203		*n* = 20,370	
	*n*	(%)	*n*	(%)	*n*	(%)	*n*	(%)	*n*	(%)
Mean age ±SD	42	±17	37	±13	45	±16	52	±17	66	±19
Age group										
18–49	169,846	69.1	125,272	80.3	31,620	61.6	8,460	46.5	4,494	22.1
50–64	50,699	20.6	26,204	16.8	14,233	27.7	5,592	30.7	4,670	22.9
65–74	12,785	5.2	3,646	2.3	3,626	7.1	2,202	12.1	3,311	16.3
75+	12,626	5.1	894	0.6	1,888	3.7	1,949	10.7	7,895	38.8
Sex										
Women	136,175	55.4	81,059	52.0	31,801	61.9	11,608	63.8	11,707	57.5
Men	109,781	44.6	74,957	48.0	19,566	38.1	6,595	36.2	8,663	42.5
Living environment										
At home	239,058	97.2	155,393	99.6	50,550	98.4	17,284	95.0	15,831	77.7
CHSLDs	2,276	0.9	94	0.1	201	0.4	264	1.5	1,717	8.4
RPAs	3,144	1.3	191	0.1	407	0.8	454	2.5	2,092	10.3
Others	1,478	0.6	338	0.2	209	0.4	201	1.1	730	3.6
Number of comorbidities										
0	156,016	63.4	156,016	100.0	0	0.0	0	0.0	0	0.0
1	51,367	20.9	0	0.0	51,367	100.0	0	0.0	0	0.0
2	18,203	7.4	0	0.0	0	0.0	18,203	100.0	0	0.0
≥3	20,370	8.3	0	0.0	0	0.0	0	0.0	20,370	100.0
Vaccination status^a^										
Unvaccinated	23,370	9.5	15,485	9.9	4,664	9.1	1,574	8.6	1,647	8.1
Vaccinated	222,586	90.5	140,531	90.1	46,703	90.9	16,629	91.4	18,723	91.9
Number of vaccine doses										
0	18,789	7.6	12,526	8.0	3,731	7.3	1,275	7.0	1,257	6.2
1	4,338	1.8	2,832	1.8	877	1.7	282	1.5	347	1.7
2	194,025	78.9	129,181	82.8	40,364	78.6	13,220	72.6	11,260	55.3
3	28,707	11.7	11,446	7.3	6,375	12.4	3,417	18.8	7,469	36.7
4+	97	0.0	31	0.0	20	0.0	9	0.0	37	0.2
Epidemiological week										
2021–49	6,615	2.7	4,041	2.6	1,514	2.9	557	3.1	503	2.5
2021–50	15,277	6.2	10,054	6.4	3,186	6.2	1,130	6.2	907	4.5
2021–51	49,321	20.1	34,390	22.0	9,603	18.7	2,990	16.4	2,338	11.5
2021–52	85,430	34.7	54,833	35.1	17,977	35.0	6,303	34.6	6,317	31.0
2022–01	89,313	36.3	52,698	33.8	19,087	37.2	7,223	39.7	10,305	50.6
Hospitalisation										
Yes	4,242	1.7	775	0.5	650	1.3	508	2.8	2,309	11.3
ICU admissions										
Yes	650	0.3	146	0.1	120	0.2	94	0.5	290	1.4
Death										
Yes	1,108	0.5	47	0.0	119	0.2	123	0.7	819	4.0

Abbreviations: CHSLD: Long-term care or nursing facilities; ICU: Intensive care unit; n/a: not available; RPA: private seniors’ residences.

^a^The vaccinated group included all individuals adequately vaccinated, i.e., all individuals who received two vaccine doses (or one Janssen vaccine dose) or a combination of these vaccines with a respected minimal interval between the 2 doses. The unvaccinated group included all other individuals.

### Hospitalizations

**Figure 1.**
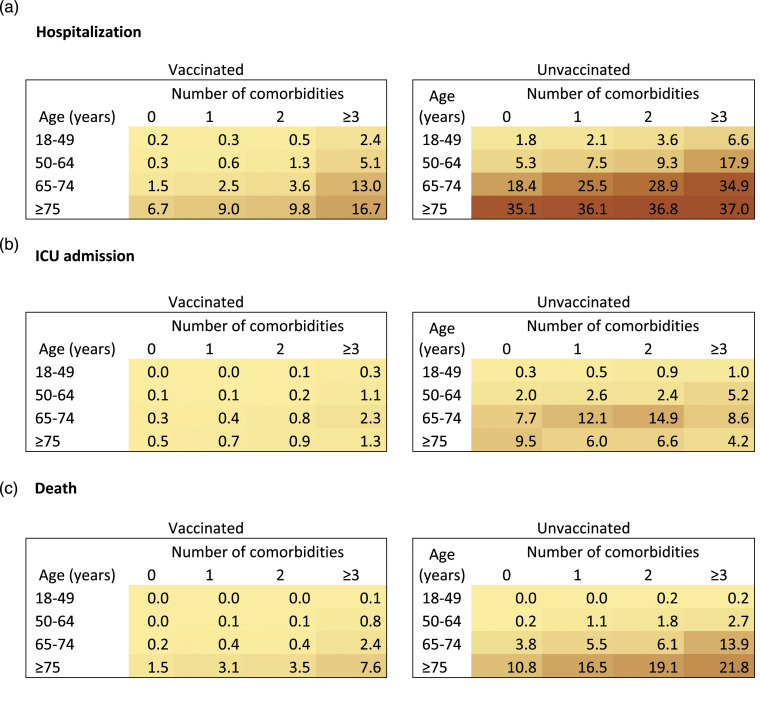
Percentage of adults with COVID-19 identified between Dec 5^th^ 2021-Jan 9^th^ 2022 with COVID-19 hospitalisation (a), intensive care unit (ICU) admission (b) or death (c) by 13^th^ February 2022 by number of comorbidities, stratified by age and vaccination status during the Omicron wave Québec, Canada (*n* = 245,956). Graduated color representation of percentage: yellow (smaller percentage), brown (higher percentage). The vaccinated group included all individuals adequately vaccinated, i.e., all individuals who received two vaccine doses (or one Janssen vaccine dose) or a combination of these vaccines with a respected minimal interval between the 2 doses. The unvaccinated group included all other individuals. Confidence intervals are reported in the supplementary file, Table A.2.

The proportion of cases with hospitalization increased for each additional comorbidity in all age groups, in both vaccinated and unvaccinated confirmed COVID-19 cases (Figure 1). Among the vaccinated cases in the 18-49 age group, the percentage of hospitalization increased from 0.2% in those without comorbidity to 2.4% in those having ≥3 comorbidities (Figure 1). Similar increases were observed in all age groups and the percentage of hospitalization reached 16.7% in the ≥3 comorbidities and ≥75 age groups. Similarly, in unvaccinated cases, the percentage of hospitalization increased when the number of comorbidities increased. The percentage of hospitalization also increased with increasing comorbidities in all age groups. Indeed, the percentage of hospitalization increased from 1.8% to 6.6% and from 35.1% to 37.0% in the 18-49 and ≥75 age groups, respectively, when the number of comorbidities increased from 0 to ≥3. Percentages in unvaccinated cases were higher than in vaccinated cases in all examined age groups.

**Figure 2.**
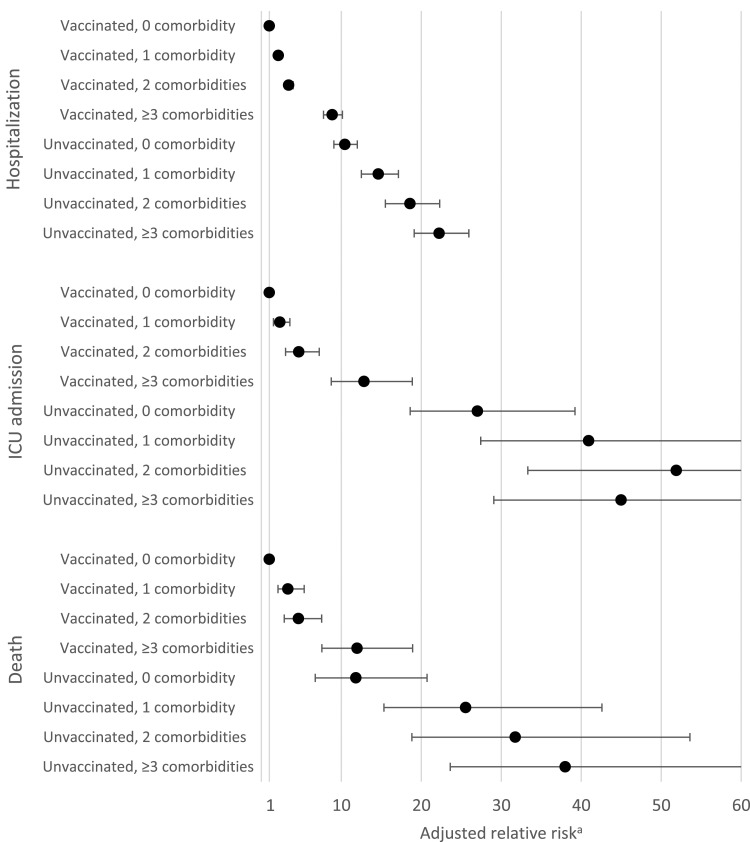
Risks of COVID-19 hospitalisation, intensive care unit admission or death according to the number of comorbidities and the vaccination status by 13^th^ February 2022 during the Omicron wave among adults with COVID-19 identified during the Omicron wave (Dec 5^th^ 2021-Jan 9^th^ 2022, Quebec, Canada) (*n* = 245,956). Abbreviation: ICU: Intensive care unit. ^a^ Adjusted for age, sex, week, living environment, material and social deprivation.

After adjustment for age and all other covariates, the risk of hospitalization (all age groups included) still increased for each additional comorbidity in both vaccinated and unvaccinated groups (Table 2, Figure 2) and the risk was consistently higher among unvaccinated. Compared with vaccinated cases without comorbidities, the risk of hospitalization was almost 9X higher (RRa=8.87; 95%CI[7.77-10.14]) in vaccinated cases with ≥3 comorbidities, but it was 22X higher (RRa=22.24; 95%CI[19.07-25.95]) in unvaccinated cases with ≥3 comorbidities. The adjusted relative risks also indicated that the risks of hospitalisation where similar between the 0 comorbidities/unvaccinated group and ≥3 comorbidities/vaccinated group.

**Table 2.** Association between the number of comorbidities and the risks of COVID-19 hospitalisation, intensive care unit admission or death by 13^th^ February 2022 while accounting for vaccination status during the Omicron wave among adults with COVID-19 identified during the Omicron wave (Dec 5^th^ 2021-Jan 9^th^ 2022, Quebec, Canada) (*n* = 245,956).

Vaccination status^a^	Number of comorbidities	Frequency		Adjusted Relative Risk	
		Event	Non-event	RRa	[IC 95%]
Hospitalization					
Vaccinated	0	368	140,163	1.00	(reference group)
	1	416	46,287	2.13	[1.85−2.46]
	2	358	16,271	3.42	[2.94−3.97]
	≥3	1,953	16,770	8.87	[7.77−10.14]
Unvaccinated	0	407	15,078	10.44	[9.07−12.01]
	1	234	4,430	14.63	[12.48−17.14]
	2	150	1,424	18.59	[15.50−22.29]
	≥3	356	1,291	22.24	[19.07−25.95]
ICU admission					
Vaccinated	0	38	140,493	1.00	(reference group)
	1	50	46,653	2.32	[1.51−3.56]
	2	51	16,578	4.68	[3.02−7.23]
	≥3	227	18,496	12.84	[8.74−18.87]
Unvaccinated	0	108	15,377	27.02	[18.61−39.22]
	1	70	4,594	40.93	[27.43−61.06]
	2	43	1,531	51.90	[33.32−80.83]
	≥3	63	1,584	44.99	[29.06−69.67]
Death					
Vaccinated	0	23	140,508	1.00	(reference group)
	1	78	46,625	3.32	[2.07−5.35]
	2	82	16,547	4.64	[2.86−7.54]
	≥3	674	18,049	11.97	[7.57−18.91]
Unvaccinated	0	24	15,461	11.82	[6.74−20.71]
	1	41	4,623	25.55	[15.32−42.59]
	2	41	1,533	31.75	[18.82−53.59]
	≥3	145	1,502	38.00	[23.62−61.14]

Abbreviation: ICU: Intensive care unit; RRa:Relative risk adjusted for age, sex, week, living environment, material and social deprivation

^a^The vaccinated group included all individuals adequately vaccinated, i.e., all individuals who received two vaccine doses (or one Janssen vaccine dose) or a combination of these vaccines with a respected minimal interval between the 2 doses. The unvaccinated group included all other individuals.

### ICU admissions

The proportion of cases with ICU admission increased for each additional comorbidity in all age groups except in the unvaccinated ≥75 age group where it gradually decreased with the number of comorbidities (Figure 1). Proportion of cases with ICU admission in unvaccinated were consistently higher than in vaccinated cases in all subgroups.

In the adjusted analysis (all age groups included), there was an association between each additional comorbidity and the risk of ICU admission in both vaccinated and unvaccinated groups (Table 2, Figure 2). Furthermore, the adjusted relative risk of ICU admission was consistently higher among unvaccinated, and we may observe that the risks were lower in the ≥3 comorbidities/vaccinated group than in the 0 comorbidities/unvaccinated group.

### Deaths

The proportion of cases with death increased for each additional comorbidity in all age groups in both vaccinated and unvaccinated cases (Figure 1). Among the vaccinated cases, the percentage of death increased from <0.1% to 0.1% and from 1.5% to 7.6% in the 18-49 and ≥75 age groups, respectively, when the number of comorbidities increased from 0 to ≥3. In unvaccinated individuals, each additional comorbidity was also associated with an increase in the percentage of death in all age groups, and percentages was systematically higher than among vaccinated cases.

After adjustment for age and all other covariates, we observed that the risk of death increased for each additional comorbidity in both vaccinated and unvaccinated groups (Table 2, Figure 2) and that the risk was consistently higher among unvaccinated cases. Compared with vaccinated cases without comorbidities, the risk of death was almost 12X higher (RRa=11.97; 95%CI[7.57-18.91]) in vaccinated cases with ≥3 comorbidities, but it was 38X higher (RRa=38.00; 95%CI[23.62-61.14]) in unvaccinated cases with ≥3 comorbidities. The adjusted relative risks also indicated that the risks of death where similar between the 0 comorbidities/unvaccinated group and ≥3 comorbidities/vaccinated group.

### Sensitivity and supplementary analysis

The proportion of cases with hospitalization, ICU admission or death stratified by number of comorbidities were very similar when restricted to the last two weeks of available data when the proportion of Omicron variant cases was greater than 90% when we included hospital-associated cases (except that we observed higher percentage of hospitalization among older adults and those with a higher number of comorbidities) (supplementary file, Tables A.3, A.4, A.7, A.8). The increase with each additional comorbidity in the percentage of hospitalization, ICU admission or death of COVID-19 cases was also observed by number of vaccine doses (supplementary file, Table A.5). In general, risk decreased with the increase of the number of vaccine dose. Estimation of relative risks by age subgroups were possible only for the hospitalisation outcome. Since interaction terms with age were not significant [for ICU admission] or model failed to converge [for death], age subgroups for both outcomes are not reported. The risk of hospitalization for each age subgroup increased for each additional comorbidity in both vaccinated and unvaccinated groups (supplementary file, Table A.9) and the risk was consistently higher among unvaccinated.

## Discussion

In this cohort study based on COVID-19 population-based surveillance data, comorbidities remained a significant risk factor of severe complications among COVID-19 cases during the Omicron wave of the pandemic. The proportion of cases with hospitalization, ICU admission, and death increased with the number of comorbidities and the impact of comorbidities appeared to be higher in unvaccinated individuals. Also, the proportion of complications in vaccinated individuals with ≥3 comorbidities were lower than in unvaccinated individuals without comorbidities.

The association we observed in this study between the number of comorbidities and the risk of hospitalization when taking into account the vaccination status is similar to that in a Danish study using surveillance data.^8^ In their study, Kahn et al. included 55,269 COVID-19 confirmed cases during the Omicron wave and observed similar associations but a lower risk of hospitalization in both vaccinated and unvaccinated groups. The higher risk of hospitalization observed in our study may be explained by a lower testing capacity in the province of Québec or COVID-19 positives cases hospitalized for non-COVID reasons, possibly more frequent among individuals with comorbidities. To our knowledge, no study has explored the association between the number of comorbidities and the risks of ICU admission or death during the Omicron wave. A pre-print paper using surveillance data of 50 Omicron hospitalized cases in France has shown a higher rate of serious hospital event (that included ICU admission or death) among cases with at least one comorbidity than among those without.^19^

In the ≥75 age group, we observed that the proportion of ICU admission was lower than in the 65-74 age group and it was decreasing with increased number of comorbidities. A similar pattern has been observed in all Canadian provinces since the pandemic outbreak.^20^ In patients with unfavourable prognosis related to underlying illnesses, a less aggressive therapeutic approach is generally adopted and in Quebec it is determined by a form specifying the predefined levels of care.^21^ The lower proportion of ICU admission in the very elderly and those with more comorbidities may be explained by the fact that higher proportion of these groups have predefined levels of care that preclude ICU admission.^22,23^

This study shows that vulnerability to COVID-19 is higher in unvaccinated individuals, even in the absence of comorbidities. Similar or even lower adjusted risks of hospitalization, ICU admission, and death in vaccinated individuals with ≥3 comorbidities than in unvaccinated individuals without comorbidities indicate a potential efficacy of adequate vaccination in later groups. Furthermore, these results suggest that vaccination may reduce the risk of complications also in individuals not seeing themselves as vulnerable.A key strength of our study is the use of a population-based surveillance database that includes all laboratory confirmed cases in the province of Quebec, Canada. Because the testing capacity was widely available and free of charge, information on a large number of confirmed COVID-19 cases was available. We only excluded cases with no information on their pre-existing medical conditions. As the characteristics of the study population is similar to all cases registered in the surveillance database, our result seemed generalizable to all confirmed COVID-19 cases (supplementary file, Table A.6). Another strength of our surveillance database is the real-time access to the vaccination status, to COVID-19 related hospitalization (including ICU admission) and COVID-19 related death. The low discrepancy between the reported COVID-19 deaths and their estimation based on overall mortality may suggest an adequate report of death information due to COVID-19 in the province of Québec which limits misclassification.^24^

A major limitation of our study is that we do not have information on the variant of the virus at the individual-level. The only information available is the weekly proportion of Omicron variant among randomly sampled cases.^7^ On the first day of our study (December 5^th^, 2021), 38% of confirmed cases were Omicron variant and by the end of the week, 73% were Omicron variant. This proportion increase to 86% on December 19^th^, 2021, and 96% by the end of the study (January 9^th^, 2022). Tests performed on 627 hospitalized patients (admitted between December 21^st^ and January 10^th^ show that the Delta variant was more present in hospitals (around 20% of tested patients); this suggests that the burden of COVID-19 on the population was still influenced in part by the Delta variant during the Omicron wave.^25^ To reduce the potential contamination of our study population by Delta cases, we performed a sensitivity analysis on confirmed cases between December 26^th^, 2021, and January 9^th^, 2022, when the proportion of Omicron variant was higher than 90%. The results were quite similar to those of the main analysis. This similarity may be explained by the fact that the majority of cases included in the study (71%) where confirmed in these last two weeks. Another limitation is that the testing capacity was under pressure due to the rapid increase in the number of cases during the Omicron wave. This may have led to an overestimation of proportion of cases with complication, especially among groups of people at lower risk of complications (younger, without pre-existing medical conditions persons), and consequently to an overestimation of the percentage point estimate in those groups and thus to an underestimation of relative risk between groups with and without comorbidities. A shorter lookback search window in administrative data file for cancer, depression and drug and alcohol abuse (5-years), as opposed to 10-years for other diseases, may also have led to an underestimation of relative risk between groups with and without comorbidities,^8^ we were unable to distinguish between hospitalization with COVID-19 as the primary cause and incidental cases admitted to hospital for reasons other than COVID-19. Unpublished data using our database show that a majority of hospitalizations during the study period were related to COVID-19 as the primary reason for admission. Patients admitted for reasons other than COVID-19 may have more comorbidities than those admitted for COVID-19. However, because comorbidities are a risk factor for COVID-19 hospitalization, the opposite may also be true. Because we lack information on the distribution of comorbidities among these 2 groups, it is difficult to assess whether our point estimates overestimate or underestimate the true association between comorbidity and hospitalization for COVID-19.

## Conclusion

This study using population-based surveillance data shows that comorbidities remain consistently associated with increased risks of hospitalization, ICU admission, and death in Omicron COVID-19 cases, similar to previous SARS-CoV2 variants, and that these risks are highest in unvaccinated individuals. Because the Omicron variant is at the moment of the submission of the manuscript the most prevalent worldwide, our results support the importance to promoting vaccination in persons with pre-existing medical conditions to reduce severe COVID-19 complications. A different study would be needed to study the impact of comorbidities on long COVID-19 in Omicron variant cases.

## Supplemental Material

Supplemental Material - Impact of chronic comorbidities on hospitalization, intensive care unit admission and death among adult vaccinated and unvaccinated COVID-19 confirmed cases during the Omicron wave Click here for additional data file. Supplemental Material for Impact of chronic comorbidities on hospitalization, intensive care unit admission and death among adult vaccinated and unvaccinated COVID-19 confirmed cases during the Omicron wave by Marc Simard, Véronique Boiteau, Élise Fortin, Sonia Jean, Louis Rochette, Pierre-Luc Trépanier and Rodica Gilca in Journal of Multimorbidity and Comorbidity
