# Supplementary material for: Impact of chronic comorbidities on hospitalization, intensive care unit admission and death among adult vaccinated and unvaccinated COVID-19 confirmed cases during the Omicron wave
Source: J Multimorb Comorb. 2023 Apr 29;13:26335565231169567. doi: 10.1177/26335565231169567 (PMC10152240; doi:10.1177/26335565231169567)
Supplement: Supplemental Material - Impact of chronic comorbidities on hospitalization, intensive care unit admission and death among adult vaccinated and unvaccinated COVID-19 confirmed cases during the Omicron wave [file sj-pdf-1-cob-10.1177_26335565231169567.pdf]

Simard M., Boiteau V, Fortin E, Jean S, Rochette L, Trépanier PL, Gilca R. Impact of chronic comorbidities on hospitalization, intensive care unit admission and death among adult vaccinated and unvaccinated COVID-19 confirmed cases during the Omicron wave, Journal of Multimorbidity and Comorbidity, 2023

Table A.1: List of 21 pre-existing comorbidities

Comorbidity	Included diseases[a]
Hypertension	Hypertension
Respiratory diseases	Chronic pulmonary disease, Pulmonary circulation disorders
Cardiovascular diseases	Cardiac arrhythmias, Peripheral vascular disorders, Myocardial infarction, Congestive heart failure, Valvular diseases, Cerebrovascular disease
Diabetes	Diabetes uncomplicated, Diabetes complicated
Cancer	Any tumor without metastasis, Metastatic cancer
Neurological disorders	Dementia, Neurological disorders
Kidney disease	Kidney disease
Liver disease	Liver disease
Immune system problem	Rheumatoid arthritis / collagen vascular disease, AIDS/HIV
Obesity	Obesity
Fluid and electrolyte disorders	Fluid and electrolyte disorders
Hypothyroidism	Hypothyroidism
Psychosis	Psychosis
Depression	Depression
Alcohol abuse	Alcohol abuse
Drug abuse	Drug abuse
Weight loss	Weight loss
Paralysis	Paralysis
Coagulopathy	Coagulopathy
Anaemia	Deficiency anemia, Blood loss anemia
Ulcer	Ulcer diseases

[a] List of ICD codes for each included disease are available elsewhere: Simard M, Sirois C, Candas B. Validation of the Combined Comorbidity Index of Charlson and Elixhauser to Predict 30-Day Mortality Across ICD-9 and ICD-10. Med Care. 2018;56(5):441-7.

Figure A.1: Study selection flowchart

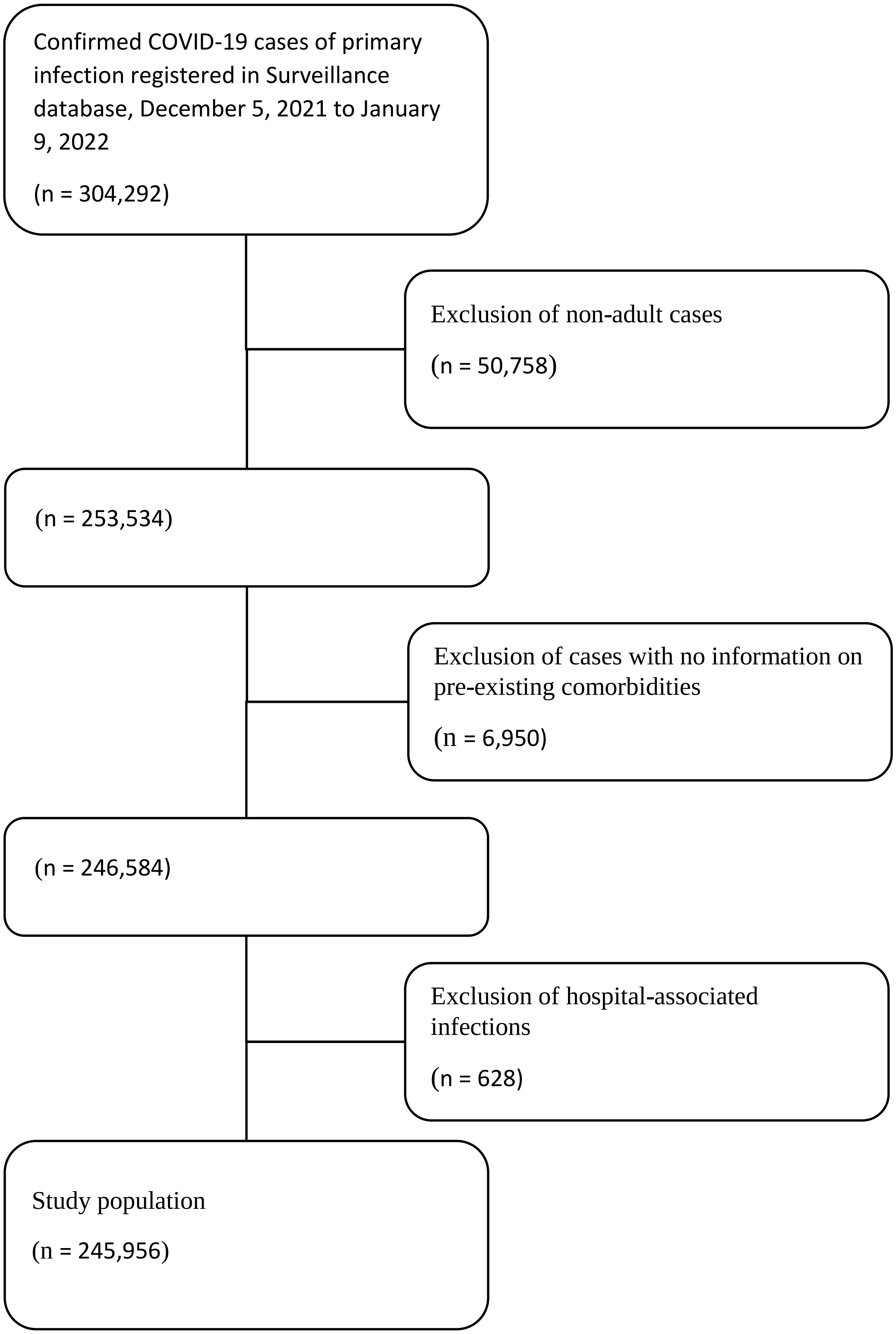

Table A.2: Percentage and 95% confidence interval of adults with COVID-19 identified between Dec 5th 2021-Jan 9th 2022 with COVID-19 hospitalisation, intensive care unit admission, or death by 13th February 2022 by number of comorbidities, stratified by age and vaccination status during the Omicron wave, Québec, Canada (n=245,956).

Age group	Number of comorbidities	Vaccinated[a]				Unvaccinated[a]			
		Event	Non-event	%	95% CI	Event	Non-event	%	95% CI
Hospitalization									
18-49	0	195	111,745	0.17	(0.15 - 0.20)	237	13,095	1.78	(1.57 - 2.02)
	1	95	28,111	0.34	(0.28 - 0.41)	73	3,341	2.14	(1.70 - 2.68)
	2	39	7,430	0.52	(0.38 - 0.71)	36	955	3.63	(2.64 - 5.01)
	≥3	92	3,813	2.36	(1.93 - 2.88)	39	550	6.62	(4.89 - 8.97)
50-64	0	67	24,319	0.27	(0.22 - 0.35)	96	1,722	5.28	(4.35 - 6.42)
	1	77	13,204	0.58	(0.46 - 0.72)	71	881	7.46	(5.96 - 9.33)
	2	66	5,193	1.25	(0.99 - 1.59)	31	302	9.31	(6.66 - 13.02)
	≥3	221	4,081	5.14	(4.52 - 5.84)	66	302	17.93	(14.41 - 22.32)
65-74	0	51	3,334	1.51	(1.15 - 1.98)	48	213	18.39	(14.24 - 23.75)
	1	86	3,375	2.48	(2.02 - 3.06)	42	123	25.45	(19.60 - 33.05)
	2	75	2,013	3.59	(2.88 - 4.49)	33	81	28.95	(21.71 - 38.59)
	≥3	402	2,700	12.96	(11.83 - 14.20)	73	136	34.93	(29.03 - 42.03)
≥75	0	55	765	6.71	(5.20 - 8.66)	26	48	35.14	(25.78 - 47.88)
	1	158	1,597	9.00	(7.76 - 10.45)	48	85	36.09	(28.79 - 45.25)
	2	178	1,635	9.82	(8.54 - 11.29)	50	86	36.76	(29.49 - 45.83)
	≥3	1,238	6,176	16.70	(15.87 - 17.57)	178	303	37.01	(32.93 - 41.58)
ICU admission									
18-49	0	10	111,930	0.01	(0.00 - 0.02)	44	13,288	0.33	(0.25 - 0.44)
	1	9	28,197	0.03	(0.02 - 0.06)	17	3,397	0.50	(0.31 - 0.80)
	2	7	7,462	0.09	(0.04 - 0.20)	9	982	0.91	(0.47 - 1.74)

Age group	Number of comorbidities	Vaccinated[a]				Unvaccinated[a]			
		Event	Non-event	%	95% CI	Event	Non-event	%	95% CI
	≥3	11	3,894	0.28	(0.16 - 0.51)	6	583	1.02	(0.46 - 2.26)
50-64	0	14	24,372	0.06	(0.03 - 0.10)	37	1,781	2.04	(1.48 - 2.80)
	1	14	13,267	0.11	(0.06 - 0.18)	25	927	2.63	(1.78 - 3.87)
	2	12	5,247	0.23	(0.13 - 0.40)	8	325	2.40	(1.21 - 4.76)
	≥3	46	4,256	1.07	(0.80 - 1.43)	19	349	5.16	(3.33 - 8.00)
65-74	0	10	3,375	0.30	(0.16 - 0.55)	20	241	7.66	(5.03 - 11.68)
	1	15	3,446	0.43	(0.26 - 0.72)	20	145	12.12	(8.04 - 18.28)
	2	16	2,072	0.77	(0.47 - 1.25)	17	97	14.91	(9.62 - 23.12)
	≥3	72	3,030	2.32	(1.85 - 2.92)	18	191	8.61	(5.54 - 13.39)
≥75	0	4	816	0.49	(0.18 - 1.30)	7	67	9.46	(4.67 - 19.14)
	1	12	1,743	0.68	(0.39 - 1.20)	8	125	6.02	(3.07 - 11.78)
	2	16	1,797	0.88	(0.54 - 1.44)	9	127	6.62	(3.52 - 12.44)
	≥3	98	7,316	1.32	(1.09 - 1.61)	20	461	4.16	(2.71 - 6.39)
Death									
18-49	0	0	111,940	<0.01	(0.00 - 0.004)	3	13,329	0.02	(0.01 - 0.07)
	1	1	28,205	<0.01	(0.00 - 0.03)	0	3,414	<0.01	(0.00 - 0.14)
	2	2	7,467	0.03	(0.01 - 0.11)	2	989	0.20	(0.05 - 0.81)
	≥3	2	3,903	0.05	(0.01 - 0.20)	1	588	0.17	(0.02 - 1.20)
50-64	0	5	24,381	0.02	(0.01 - 0.05)	3	1,815	0.17	(0.05 - 0.51)
	1	8	13,273	0.06	(0.03 - 0.12)	10	942	1.05	(0.57 - 1.95)
	2	7	5,252	0.13	(0.06 - 0.28)	6	327	1.80	(0.82 - 3.98)
	≥3	34	4,268	0.79	(0.57 - 1.10)	10	358	2.72	(1.47 - 5.01)
65-74	0	6	3,379	0.18	(0.08 - 0.39)	10	251	3.83	(2.09 - 7.04)
	1	14	3,447	0.40	(0.24 - 0.68)	9	156	5.45	(2.89 - 10.30)

Age group	Number of comorbidities	Vaccinated[a]				Unvaccinated[a]			
		Event	Non-event	%	95% CI	Event	Non-event	%	95% CI
	2	9	2,079	0.43	(0.22 - 0.83)	7	107	6.14	(3.00 - 12.59)
	≥3	74	3,028	2.39	(1.90 - 2.99)	29	180	13.88	(9.90 - 19.45)
≥75	0	12	808	1.46	(0.83 - 2.57)	8	66	10.81	(5.62 - 20.80)
	1	55	1,700	3.13	(2.42 - 4.06)	22	111	16.54	(11.29 - 24.23)
	2	64	1,749	3.53	(2.78 - 4.49)	26	110	19.12	(13.53 - 27.01)
	≥3	564	6,850	7.61	(7.03 - 8.24)	105	376	21.83	(18.43 - 25.85)

Abbreviations: CI: confidence interval; ICU: Intensive care unit

[a] The vaccinated group included all individuals adequately vaccinated, i.e., all individuals who received two vaccine doses (or one Janssen vaccine dose) or a combination of these vaccines with a respected minimal interval between the 2 doses. The unvaccinated group included all other individuals.

Table A.3: Percentage and 95% confidence interval of adults with COVID-19 identified between Dec 26th 2021-Jan 9th 2022 with COVID-19 hospitalisation, intensive care unit admission, or death by 13th February 2022 by number of comorbidities, stratified by age and vaccination status during the Omicron wave, Québec, Canada (n=174,743).

Age group	Number of comorbidities	Vaccinated[a]		Unvaccinated[a]	
		%	95% CI	%	95% CI
Hospitalization					
18-49	0	0.20	(0.17 - 0.23)	1.79	(1.54 - 2.09)
	1	0.39	(0.32 - 0.49)	2.12	(1.61 - 2.78)
	2	0.50	(0.34 - 0.72)	3.53	(2.39 - 5.24)
	≥3	2.54	(2.04 - 3.17)	6.35	(4.44 - 9.09)
50-64	0	0.30	(0.23 - 0.40)	4.25	(3.27 - 5.52)
	1	0.50	(0.38 - 0.66)	6.26	(4.65 - 8.42)
	2	1.31	(1.00 - 1.71)	7.56	(4.78 - 11.93)
	≥3	5.40	(4.70 - 6.20)	19.78	(15.58 - 25.12)
65-74	0	1.83	(1.36 - 2.46)	18.82	(13.96 - 25.36)
	1	2.64	(2.07 - 3.35)	26.13	(19.11 - 35.72)
	2	3.81	(2.95 - 4.91)	29.87	(21.21 - 42.06)
	≥3	13.33	(12.03 - 14.75)	30.32	(23.89 - 38.50)
≥75	0	7.28	(5.50 - 9.65)	33.90	(23.74 - 48.41)
	1	9.59	(8.15 - 11.29)	37.62	(29.27 - 48.36)
	2	10.47	(9.01 - 12.17)	34.91	(26.91 - 45.27)
	≥3	16.28	(15.40 - 17.20)	34.66	(30.30 - 39.65)
ICU admission					
18-49	0	0.01	(0.00 - 0.02)	0.26	(0.17 - 0.39)
	1	0.03	(0.01 - 0.06)	0.47	(0.26 - 0.84)
	2	0.07	(0.03 - 0.20)	0.88	(0.40 - 1.95)
	≥3	0.23	(0.11 - 0.48)	0.67	(0.22 - 2.08)
50-64	0	0.07	(0.04 - 0.11)	1.26	(0.77 - 2.05)
	1	0.09	(0.05 - 0.17)	2.44	(1.50 - 3.96)
	2	0.22	(0.12 - 0.43)	1.78	(0.67 - 4.70)
	≥3	1.15	(0.84 - 1.56)	4.98	(2.99 - 8.30)
65-74	0	0.34	(0.17 - 0.68)	7.94	(4.88 - 12.90)
	1	0.36	(0.19 - 0.70)	11.30	(6.77 - 18.86)
	2	0.47	(0.22 - 0.98)	12.82	(7.19 - 22.87)
	≥3	2.03	(1.54 - 2.68)	5.00	(2.54 - 9.82)
≥75	0	0.65	(0.24 - 1.72)	7.94	(3.42 - 18.40)
	1	0.59	(0.29 - 1.17)	8.33	(4.46 - 15.58)
	2	0.76	(0.42 - 1.36)	6.54	(3.20 - 13.39)
	≥3	1.20	(0.96 - 1.50)	3.99	(2.50 - 6.36)

Age group	Number of comorbidities	Vaccinated[a]		Unvaccinated[a]	
		%	95% CI	%	95% CI
Death					
18-49	0	<0.01	(0.00 - 0.01)	0.03	(0.01 - 0.10)
	1	0.01	(0.00 - 0.04)	<0.01	(0.00 - 0.20)
	2	0.04	(0.01 - 0.15)	0.15	(0.02 - 1.04)
	≥3	0.06	(0.01 - 0.40)	<0.01	(0.00 – 1.04)
50-64	0	0.03	(0.01 - 0.07)	0.16	(0.04 - 0.63)
	1	0.05	(0.02 - 0.12)	0.61	(0.23 - 1.62)
	2	0.12	(0.05 - 0.30)	1.78	(0.67 - 4.70)
	≥3	0.75	(0.51 - 1.09)	3.30	(1.73 - 6.27)
65-74	0	0.21	(0.09 - 0.51)	4.30	(2.18 - 8.47)
	1	0.45	(0.25 - 0.80)	2.70	(0.89 - 8.25)
	2	0.33	(0.14 - 0.80)	5.19	(2.00 - 13.49)
	≥3	2.37	(1.83 - 3.06)	12.26	(8.04 - 18.68)
≥75	0	1.94	(1.11 - 3.40)	6.78	(2.63 - 17.46)
	1	3.29	(2.47 - 4.39)	17.82	(11.72 - 27.09)
	2	4.06	(3.16 - 5.22)	19.81	(13.51 - 29.06)
	≥3	7.62	(7.00 - 8.30)	21.20	(17.55 - 25.60)

Abbreviations: CI: confidence interval; ICU: Intensive care unit

[a] The vaccinated group included all individuals adequately vaccinated, i.e., all individuals who received two vaccine doses (or one Janssen vaccine dose) or a combination of these vaccines with a respected minimal interval between the 2 doses. The unvaccinated group included all other individuals.

Table A.4: Percentage and 95% confidence interval of adults with COVID-19 (including hospital-associated infections cases) identified between Dec 5th 2021-Jan 9th 2022 with COVID-19 hospitalisation, intensive care unit admission, or death by 13th February 2022 by number of comorbidities, stratified by age and vaccination status (n=246,584).

Age group	Number of comorbidities	Vaccinated[a]		Unvaccinated[a]	
		%	95% CI	%	95% CI
Hospitalization[b]					
18-49	0	0.18	(0.16 - 0.21)	1.82	(1.61 - 2.06)
	1	0.34	(0.28 - 0.41)	2.17	(1.73 - 2.71)
	2	0.59	(0.44 - 0.79)	3.83	(2.80 - 5.23)
	≥3	2.70	(2.24 - 3.26)	7.25	(5.44 - 9.67)
50-64	0	0.31	(0.25 - 0.39)	5.28	(4.35 - 6.42)
	1	0.64	(0.52 - 0.79)	7.56	(6.05 - 9.43)
	2	1.40	(1.12 - 1.76)	9.58	(6.89 - 13.32)
	≥3	5.82	(5.16 - 6.56)	19.68	(16.05 - 24.14)
65-74	0	1.74	(1.35 - 2.24)	19.32	(15.10 - 24.72)
	1	3.10	(2.58 - 3.73)	27.22	(21.27 - 34.83)
	2	4.28	(3.50 - 5.24)	29.57	(22.30 - 39.20)
	≥3	14.75	(13.56 - 16.03)	36.45	(30.54 - 43.50)
≥75	0	9.36	(7.59 - 11.55)	38.46	(29.05 - 50.93)
	1	11.47	(10.09 - 13.04)	39.29	(31.97 - 48.27)
	2	11.95	(10.57 - 13.53)	37.68	(30.40 - 46.70)
	≥3	19.35	(18.49 - 20.26)	40.47	(36.42 - 44.97)
ICU admission[c]					
18-49	0	0.01	(0.00 - 0.02)	0.33	(0.25 - 0.44)
	1	0.03	(0.02 - 0.06)	0.50	(0.31 - 0.80)
	2	0.09	(0.04 - 0.20)	0.91	(0.47 - 1.74)
	≥3	0.33	(0.19 - 0.57)	1.01	(0.46 - 2.24)
50-64	0	0.06	(0.04 - 0.10)	2.04	(1.48 - 2.80)
	1	0.11	(0.06 - 0.18)	2.73	(1.87 - 3.99)
	2	0.27	(0.16 - 0.45)	2.40	(1.21 - 4.75)
	≥3	1.11	(0.84 - 1.47)	5.05	(3.26 - 7.83)
65-74	0	0.38	(0.22 - 0.66)	7.58	(4.97 - 11.55)
	1	0.49	(0.30 - 0.78)	12.43	(8.33 - 18.54)
	2	0.81	(0.50 - 1.30)	14.78	(9.53 - 22.93)
	≥3	2.37	(1.89 - 2.96)	8.41	(5.41 - 13.09)
≥75	0	0.59	(0.25 - 1.42)	8.97	(4.43 - 18.19)
	1	0.67	(0.38 - 1.17)	7.14	(3.93 - 12.98)

Age group	Number of comorbidities	Vaccinated[a]		Unvaccinated[a]	
		%	95% CI	%	95% CI
	2	0.92	(0.57 - 1.47)	6.52	(3.47 - 12.27)
	≥3	1.36	(1.12 - 1.64)	4.13	(2.71 - 6.27)
Death					
18-49	0	<0.01	(0.00 - 0.004)	0.02	(0.01 - 0.07)
	1	<0.01	(0.00 - 0.03)	<0.01	(0.00 - 0.14)
	2	0.03	(0.01 - 0.11)	0.20	(0.05 - 0.80)
	≥3	0.05	(0.01 - 0.20)	0.17	(0.02 - 1.20)
50-64	0	0.02	(0.01 - 0.05)	0.17	(0.05 - 0.51)
	1	0.06	(0.03 - 0.12)	1.05	(0.57 - 1.94)
	2	0.21	(0.12 - 0.38)	1.80	(0.81 - 3.97)
	≥3	0.83	(0.60 - 1.15)	2.66	(1.44 - 4.90)
65-74	0	0.27	(0.14 - 0.51)	3.79	(2.06 - 6.96)
	1	0.46	(0.28 - 0.75)	5.92	(3.24 - 10.79)
	2	0.48	(0.26 - 0.88)	6.09	(2.97 - 12.48)
	≥3	2.65	(2.15 - 3.28)	13.55	(9.66 - 19.01)
≥75	0	1.66	(0.99 - 2.79)	10.26	(5.32 - 19.77)
	1	3.33	(2.59 - 4.27)	16.43	(11.31 - 23.87)
	2	3.66	(2.90 - 4.62)	19.57	(13.95 - 27.44)
	≥3	8.24	(7.65 - 8.88)	21.61	(18.32 - 25.50)

Abbreviations: CI: confidence interval; ICU: Intensive care unit

[a] The vaccinated group included all individuals adequately vaccinated, i.e., all individuals who received two vaccine doses (or one Janssen vaccine dose) or a combination of these vaccines with a respected minimal interval between the 2 doses. The unvaccinated group included all other individuals.

[b] Index hospitalisation when the cases have been potentially infected at hospital (nosocomial case) are included in the numerator

[c] ICU admission occurring during the index hospitalisation are included in the numerator

Table A.5 Percentage and 95% confidence interval of adults with COVID-19 identified between Dec 5th 2021-Jan 9th 2022 with COVID-19 hospitalisation, intensive care unit admission, or death by 13th February 2022 by number of comorbidities, stratified by age and number of vaccine doses during the Omicron wave, Québec, Canada (n=245,816).

Age group	Number of comorbidities	0 vaccine dose		1 vaccine dose[a]		2 vaccine doses		≥3 vaccine doses[b]	
		%	95% CI	%	95% CI	%	95% CI	%	95% CI
Hospitalization									
18-49	0	2.01	(1.76 - 2.29)	0.91	(0.60 - 1.37)	0.17	(0.14 - 0.19)	0.26	(0.17 - 0.41)
	1	2.26	(1.76 - 2.90)	1.82	(1.04 - 3.19)	0.34	(0.28 - 0.42)	0.30	(0.15 - 0.61)
	2	4.30	(3.10 - 5.98)	1.06	(0.27 - 4.20)	0.54	(0.39 - 0.75)	0.34	(0.11 - 1.06)
	≥3	7.47	(5.41 - 10.32)	4.13	(1.75 - 9.75)	1.92	(1.51 - 2.45)	4.79	(3.33 - 6.87)
50-64	0	5.99	(4.91 - 7.30)	1.18	(0.38 - 3.64)	0.30	(0.23 - 0.38)	0.16	(0.07 - 0.38)
	1	8.74	(6.96 - 10.97)	1.95	(0.64 - 5.97)	0.55	(0.43 - 0.70)	0.66	(0.38 - 1.13)
	2	9.61	(6.71 - 13.75)	8.16	(3.19 - 20.88)	1.12	(0.85 - 1.48)	1.90	(1.19 - 3.04)
	≥3	20.14	(15.97 - 25.40)	12.16	(6.59 - 22.44)	4.72	(4.05 - 5.49)	6.51	(5.14 - 8.25)
65-74	0	20.00	(15.44 - 25.90)	6.90	(1.81 - 26.27)	1.72	(1.28 - 2.29)	0.79	(0.35 - 1.74)
	1	28.28	(21.82 - 36.64)	5.88	(0.88 - 39.39)	2.46	(1.92 - 3.13)	2.56	(1.71 - 3.83)
	2	29.70	(22.00 - 40.10)	18.18	(5.19 - 63.69)	4.04	(3.15 - 5.19)	2.70	(1.69 - 4.32)
	≥3	39.49	(32.54 - 47.93)	24.44	(14.62 - 40.86)	14.10	(12.65 - 15.73)	10.90	(9.23 - 12.87)
≥75	0	36.07	(25.82 - 50.37)	36.36	(16.64 - 79.47)	8.79	(6.46 - 11.96)	4.49	(2.86 - 7.05)
	1	39.81	(31.57 - 50.21)	20.00	(9.13 - 43.80)	11.73	(9.77 - 14.09)	6.38	(4.96 - 8.20)
	2	42.72	(34.16 - 53.42)	16.13	(7.23 - 35.99)	14.63	(12.36 - 17.32)	6.22	(4.91 - 7.89)
	≥3	40.88	(36.12 - 46.28)	28.43	(20.90 - 38.68)	26.61	(24.97 - 28.35)	11.23	(10.37 - 12.16)
ICU admission									
18-49	0	0.39	(0.29 - 0.53)	0.08	(0.02 - 0.33)	0.01	(0.01 - 0.02)	<0.01	(0.00 - 0.06)
	1	0.59	(0.36 - 0.97)	0.15	(0.02 - 1.08)	0.03	(0.01 - 0.06)	0.08	(0.02 - 0.30)
	2	1.14	(0.59 - 2.18)	<0.01	(0.00 – 2.40)	0.11	(0.05 - 0.22)	<0.01	(0.00 - 0.53)
	≥3	1.32	(0.60 - 2.92)	<0.01	(0.00 – 3.70)	0.21	(0.10 - 0.44)	0.68	(0.26 - 1.82)

Age group	Number of comorbidities	0 vaccine dose		1 vaccine dose[a]		2 vaccine doses		≥3 vaccine doses[b]	
		%	95% CI	%	95% CI	%	95% CI	%	95% CI
50-64	0	2.34	(1.70 - 3.24)	0.39	(0.06 - 2.78)	0.06	(0.04 - 0.11)	0.03	(0.00 - 0.23)
	1	2.96	(1.98 - 4.42)	1.30	(0.33 - 5.15)	0.11	(0.06 - 0.19)	0.10	(0.03 - 0.40)
	2	2.85	(1.44 - 5.64)	<0.01	(0.00 - 8.68)	0.18	(0.09 - 0.37)	0.45	(0.17 - 1.19)
	≥3	6.36	(4.07 - 9.95)	1.35	(0.19 - 9.47)	1.02	(0.73 - 1.43)	1.22	(0.70 - 2.14)
65-74	0	8.26	(5.37 - 12.71)	3.45	(0.50 - 23.66)	0.34	(0.18 - 0.66)	0.13	(0.02 - 0.93)
	1	13.79	(9.18 - 20.72)	<0.01	(0.00 - 21.63)	0.47	(0.27 - 0.82)	0.33	(0.11 - 1.03)
	2	14.85	(9.31 - 23.69)	9.09	(1.40 - 58.91)	0.89	(0.52 - 1.53)	0.64	(0.24 - 1.69)
	≥3	10.83	(6.91 - 16.96)	2.22	(0.32 - 15.43)	2.84	(2.19 - 3.68)	1.41	(0.86 - 2.29)
≥75	0	11.48	(5.72 - 23.04)	<0.01	(0.00 - 30.02)	0.95	(0.36 - 2.52)	<0.01	(0.00 - 1.14)
	1	7.41	(3.80 - 14.43)	<0.01	(0.00 - 15.76)	0.93	(0.47 - 1.85)	0.45	(0.17 - 1.19)
	2	8.74	(4.68 - 16.31)	<0.01	(0.00 - 13.09)	1.27	(0.69 - 2.36)	0.58	(0.26 - 1.29)
	≥3	4.70	(2.95 - 7.47)	2.94	(0.96 - 8.97)	2.62	(2.08 - 3.31)	0.60	(0.42 - 0.87)
Death									
18-49	0	0.03	(0.01 - 0.09)	<0.01	(0.00 - 0.19)	<0.01	(0.00 - 0.004)	<0.01	(0.00 - 0.06)
	1	<0.01	(0.00 - 0.17)	<0.01	(0.00 - 0.70)	<0.01	(0.00 - 0.03)	<0.01	(0.00 - 0.18)
	2	0.25	(0.06 - 1.01)	<0.01	(0.00 - 2.40)	0.02	(0.00 - 0.11)	0.11	(0.02 - 0.81)
	≥3	0.22	(0.03 - 1.56)	<0.01	(0.00 - 3.70)	0.03	(0.00 - 0.21)	0.17	(0.02 - 1.21)
50-64	0	0.20	(0.06 - 0.60)	<0.01	(0.00 - 1.79)	0.02	(0.01 - 0.05)	0.03	(0.00 - 0.23)
	1	1.29	(0.69 - 2.38)	<0.01	(0.00 - 2.93)	0.07	(0.04 - 0.14)	<0.01	(0.00 - 0.23)
	2	1.78	(0.75 - 4.24)	2.04	(0.29 - 14.20)	0.05	(0.01 - 0.18)	0.56	(0.23 - 1.34)
	≥3	3.18	(1.67 - 6.05)	1.35	(0.19 - 9.47)	0.51	(0.32 - 0.82)	1.73	(1.08 - 2.77)
65-74	0	3.91	(2.06 - 7.42)	3.45	(0.50 - 23.66)	0.15	(0.06 - 0.41)	0.26	(0.07 - 1.05)
	1	6.21	(3.30 - 11.69)	<0.01	(0.00 - 21.63)	0.39	(0.21 - 0.72)	0.45	(0.17 - 1.18)
	2	6.93	(3.39 - 14.16)	<0.01	(0.00 - 30.02)	0.27	(0.10 - 0.73)	0.79	(0.33 - 1.90)
	≥3	15.29	(10.58 - 22.09)	11.11	(4.86 - 25.39)	2.49	(1.89 - 3.28)	2.20	(1.49 - 3.24)

Age group	Number of comorbidities	0 vaccine dose		1 vaccine dose[a]		2 vaccine doses		≥3 vaccine doses[b]	
		%	95% CI	%	95% CI	%	95% CI	%	95% CI
≥75	0	13.11	(6.87 - 25.02)	<0.01	(0.00 - 30.02)	1.90	(0.96 - 3.77)	1.00	(0.38 - 2.64)
	1	17.59	(11.70 - 26.46)	12.00	(4.15 - 34.69)	4.30	(3.14 - 5.89)	2.01	(1.27 - 3.18)
	2	18.45	(12.29 - 27.69)	22.58	(11.77 - 43.33)	4.07	(2.90 - 5.72)	3.11	(2.21 - 4.37)
	≥3	24.59	(20.53 - 29.45)	15.69	(10.00 - 24.60)	10.68	(9.56 - 11.93)	5.90	(5.27 - 6.60)

Abbreviations: CI: confidence interval; ICU: Intensive care unit
[a] Cases with 1 Janssen vaccine dose are excluded from the one vaccine dose group.
[b] 97 cases have ≥4 vaccine doses

Table A.6: Description of adults with COVID-19 identified between Dec 5th 2021-Jan 9th 2022 in the study population with available information on pre-existing medical conditions (n=245,956) and in the surveillance database (n=253,534) during the Omicron wave, Québec, Canada

Variable	Study population n = 245,956		All confirmed cases in the surveillance database n = 253,534	
	n	(%)	n	(%)
Mean age; mean ±SD	42	±17	42	±17
Age group				
18-49	169,846	69.1	176,165	69.5
50-64	50,699	20.6	51,291	20.2
65-74	12,785	5.2	13,006	5.1
75+	12,626	5.1	13,072	5.2
Sex				
Women	136,175	55.4	139,235	54.9
Men	109,781	44.6	114,299	45.1
Living environment				
At home	239,058	97.2	246,416	97.2
CHSLDs	2,276	0.9	2,319	0.9
RPAs	3,144	1.3	3,230	1.3
Others	1,478	0.6	1,569	0.6
Number of vaccine doses				
0	18,789	7.6	20,531	8.1
1	4,338	1.8	4,576	1.8
2	194,025	78.9	199,210	78.6
3	28,707	11.7	29,119	11.5
4+	97	0.0	98	0.0
CDC week				
2021-49	6,615	2.7	6,824	2.7
2021-50	15,277	6.2	15,773	6.2
2021-51	49,321	20.1	50,716	20.0
2021-52	85,430	34.7	87,816	34.6
2022-01	89,313	36.3	92,405	36.4
Hospitalisation				
Yes	4,242	1.7	4,941	1.9
ICU admissions				
Yes	650	0.3	688	0.3
Death				
Yes	1,108	0.5	1,222	0.5

Abbreviations: CDC: Centers for Disease Control and Prevention; CHSLD: Long-term care or nursing facilities; ICU: Intensive care unit; RPA: private seniors' residences.

Table A.7: Association between the number of comorbidities and the risks of COVID-19 hospitalisation, intensive care unit admission or death by 13th February 2022 while accounting for vaccination status during the Omicron wave among adults with COVID-19 identified during the Omicron wave (Dec 26th 2021-Jan 9th 2022, Quebec, Canada) (n=174,743)

Vaccination status[a]	Number of comorbidities	Frequency		Adjusted Relative Risk	
		Event	Non-event	RRa	[IC95%]
Hospitalization					
Vaccinated	0	293	96,528	1.00	(reference group)
	1	325	33,510	2.04	[1.74 - 2.39]
	2	289	12,150	3.30	[2.79 - 3.91]
	≥3	1,632	13,720	8.17	[7.02 - 9.50]
Unvaccinated	0	274	10,436	8.91	[7.57 - 10.49]
	1	158	3,071	12.81	[10.64 - 15.41]
	2	101	986	15.96	[12.88 - 19.76]
	≥3	268	1,002	19.35	[16.22 - 23.09]
ICU admission					
Vaccinated	0	29	96,792	1.00	(reference group)
	1	31	33,804	1.80	[1.08 - 3.56]
	2	31	12,408	3.48	[2.05 - 7.23]
	≥3	173	15,179	10.79	[6.85 - 18.87]
Unvaccinated	0	60	10,650	20.97	[13.43 - 39.22]
	1	45	3,184	37.15	[23.20 - 61.06]
	2	27	1,060	45.25	[26.42 - 80.83]
	≥3	41	1,229	34.21	[20.22 - 69.67]
Death					
Vaccinated	0	22	96,799	1.00	(reference group)
	1	62	33,773	2.63	[1.59 - 4.32]
	2	71	12,368	3.83	[2.31 - 6.35]
	≥3	574	14,778	8.74	[5.43 - 14.08]
Unvaccinated	0	17	10,693	8.97	[4.82 - 16.69]
	1	25	3,204	16.36	[9.28 - 28.83]
	2	30	1,057	23.40	[13.33 - 41.08]
	≥3	113	1,157	26.57	[16.11 - 43.84]

Abbreviation: ICU: Intensive care unit; RRa: Relative risk adjusted for age, sex, week, living environment, material and social deprivation

[a] The vaccinated group included all individuals adequately vaccinated, i.e., all individuals who received two vaccine doses (or one Janssen vaccine dose) or a combination of these vaccines with a respected minimal interval between the 2 doses. The unvaccinated group included all other individuals.

Table A.8: Association between the number of comorbidities and the risks of COVID-19 hospitalisation, intensive care unit admission or death by 13th February 2022 while accounting for vaccination status during the Omicron wave among adults with COVID-19 (including hospital-associated infections cases) identified during the Omicron wave (Dec 5th 2021-Jan 9th 2022, Quebec, Canada) (n=246,584)

Vaccination status[a]	Number of comorbidities	Frequency		Adjusted Relative Risk	
		Event	Non-event	RRa	[IC95%]
**Hospitalization**					
Vaccinated	0	293	96,528	1.00	(reference group)
	1	325	33,510	2.04	[1.74 - 2.39]
	2	289	12,150	3.30	[2.79 - 3.91]
	≥3	1,632	13,720	8.17	[7.02 - 9.50]
Unvaccinated	0	274	10,436	8.91	[7.57 - 10.49]
	1	158	3,071	12.81	[10.64 - 15.41]
	2	101	986	15.96	[12.88 - 19.76]
	≥3	268	1,002	19.35	[16.22 - 23.09]
**ICU admission**					
Vaccinated	0	29	96,792	1.00	(reference group)
	1	31	33,804	1.80	[1.08 - 3.56]
	2	31	12,408	3.48	[2.05 - 7.23]
	≥3	173	15,179	10.79	[6.85 - 18.87]
Unvaccinated	0	60	10,650	20.97	[13.43 - 39.22]
	1	45	3,184	37.15	[23.20 - 61.06]
	2	27	1,060	45.25	[26.42 - 80.83]
	≥3	41	1,229	34.21	[20.22 - 69.67]
**Death**					
Vaccinated	0	22	96,799	1.00	(reference group)
	1	62	33,773	2.63	[1.59 - 4.32]
	2	71	12,368	3.83	[2.31 - 6.35]
	≥3	574	14,778	8.74	[5.43 - 14.08]
Unvaccinated	0	17	10,693	8.97	[4.82 - 16.69]
	1	25	3,204	16.36	[9.28 - 28.83]
	2	30	1,057	23.40	[13.33 - 41.08]
	≥3	113	1,157	26.57	[16.11 - 43.84]

Abbreviation: ICU: Intensive care unit; RRa: Relative risk adjusted for age, sex, week, living environment, material and social deprivation

[a] The vaccinated group included all individuals adequately vaccinated, i.e., all individuals who received two vaccine doses (or one Janssen vaccine dose) or a combination of these vaccines with a respected minimal interval between the 2 doses. The unvaccinated group included all other individuals.

Table A.9: Association between the number of comorbidities and the risks of COVID-19 hospitalisation by 13th February 2022 by age subgroup while accounting for vaccination status during the Omicron wave among adults with COVID-19 identified during the Omicron wave (Dec 5th 2021-Jan 9th 2022, Quebec, Canada) (n=245,956)

Vaccination status[a]	Number of comorbidities	Frequency		Adjusted Relative Risk	
		Event	Non-event	RRa	[IC95%]
18-49 years					
Vaccinated	0	195	111,745	1.00	(reference group)
	1	95	28,111	1.99	[1.55 - 2.54]
	2	39	7,430	3.09	[2.19 - 4.36]
	≥3	92	3,813	13.67	[10.67 - 17.51]
Unvaccinated	0	237	13,095	9.53	[7.89 - 11.52]
	1	73	3,341	11.97	[9.15 - 15.65]
	2	36	955	20.39	[14.36 - 28.96]
	≥3	39	550	35.85	[25.66 - 50.08]
50-64 years					
Vaccinated	0	67	24,319	1.00	(reference group)
	1	77	13,204	2.11	[1.52 - 2.93]
	2	66	5,193	4.56	[3.25 - 6.40]
	≥3	221	4,081	18.44	[14.06 - 24.20]
Unvaccinated	0	96	1,722	18.17	[13.34 - 24.74]
	1	71	881	26.08	[18.79 - 36.19]
	2	31	302	32.87	[21.76 - 49.64]
	≥3	66	302	60.97	[44.01 - 84.46]
65-74 years					
Vaccinated	0	51	3,334	1.00	(reference group)
	1	86	3,375	1.65	[1.17 - 2.32]
	2	75	2,013	2.38	[1.67 - 3.38]
	≥3	402	2,700	8.71	[6.53 - 11.61]
Unvaccinated	0	48	213	11.55	[7.93 - 16.82]
	1	42	123	16.23	[11.08 - 23.75]
	2	33	81	18.63	[12.54 - 27.68]
	≥3	73	136	23.32	[16.78 - 32.42]
≥75 years					
Vaccinated	0	55	765	1.00	(reference group)
	1	158	1,597	1.37	[1.02 - 1.84]
	2	178	1,635	1.57	[1.18 - 2.11]
	≥3	1,238	6,176	2.87	[2.21 - 3.73]
Unvaccinated	0	26	48	5.14	[3.43 - 7.69]
	1	48	85	5.45	[3.89 - 7.65]
	2	50	86	5.63	[4.04 - 7.85]

≥3	178	303	6.10	[4.62 - 8.07]

Abbreviation: ICU: Intensive care unit; RRa: Relative risk adjusted for age, sex, week, living environment, material and social deprivation

[a] The vaccinated group included all individuals adequately vaccinated, i.e., all individuals who received two vaccine doses (or one Janssen vaccine dose) or a combination of these vaccines with a respected minimal interval between the 2 doses. The unvaccinated group included all other individuals.
